# Improved LEACH Protocol Based on Underwater Energy Propagation Model, Parallel Transmission, and Replication Computing for Underwater Acoustic Sensor Networks

**DOI:** 10.3390/s24020556

**Published:** 2024-01-16

**Authors:** Kun Tian, Chang Zhou, Jun Zhang

**Affiliations:** School of Electronic and Information Engineering, South China University of Technology, Guangzhou 510641, China; a1067552189@163.com (K.T.);

**Keywords:** underwater acoustic sensor network, clustering, LEACH protocol, underwater energy propagation model, parallel transmission, replication computing

## Abstract

Underwater acoustic sensor networks (UASNs) are critical to a range of applications from oceanographic data collection to submarine surveillance. In these networks, efficient energy management is critical due to the limited power resources of underwater sensors. The LEACH protocol, a popular cluster-based protocol, has been widely used in UASNs to minimize energy consumption. Despite its widespread use, the conventional LEACH protocol faces challenges such as an unoptimized cluster number and low transmission efficiency, which hinder its performance. This paper proposes an improved LEACH protocol for cluster-based UASNs, where the cluster number is optimized with an underwater energy propagation model to reduce energy consumption, and a transmission scheduling algorithm is also employed to achieve conflict-free parallel data transmission. Replication computing is introduced to the LEACH protocol to reduce the signaling in the clustering and data transmission phases. The simulation results show that the proposed protocol outperforms several conventional methods in terms of normalized average residual energy, average number of surviving nodes, average round when the first death node occurs, and the number of packets received by the base station.

## 1. Introduction

Underwater acoustic sensor networks (UASNs) have gained much attention in recent years due to their diversity of aquatic applications, such as monitoring, auxiliary navigation, unmanned exploration or surveillance, and mine reconnaissance [1,2,3]. Underwater acoustic channels are characterized by limited bandwidth, high and variable propagation delay, time-varying multi-pathing, and fading, which leads to high bit error rates and temporary losses of connectivity [4]. In addition, sensor nodes are mainly powered by batteries that are difficult to replace, making it hard to replenish the energy of the sensor nodes. So, constructing low-energy-consumption UASNs is extremely challenging.

Clustering is a common technique that can increase the scalability of a network while reducing its energy consumption. Typical cluster-based protocols in wireless sensor networks include low-energy adaptive clustering hierarchy (LEACH) [5], hybrid energy-efficient distributed clustering (HEED) [6], power-efficient and adaptive clustering hierarchy (PEACH) [7], distributed energy-efficient clustering (DEEC) [8], etc. Among them, the LEACH protocol is one of the most classical cluster-based protocols and has been widely used in UASNs. In the LEACH protocol, a round of transmission consists of two consecutive phases, i.e., the clustering phase and the transmission phase. It can achieve energy equalization and reduce the network energy consumption effectively by periodically selecting cluster heads and re-clustering the sensor nodes [5]. However, the existing LEACH protocol for UASNs still has some shortcomings. First, cluster numbers are usually determined empirically in the clustering phase, which is not optimal in many cases. Second, the transmission efficiency is limited due to the low parallelism of the cluster head node and sensor node data transmission. Third, the signaling in the clustering and transmission phases should be reduced to lower the energy consumption and improve the clustering and transmission efficiency.

In this paper, we propose an improved LEACH protocol based on the underwater energy propagation model, parallel transmission, and replication computing for cluster-based UASNs. The contributions of our work include the following:

- A cluster number optimization method based on the underwater energy propagation model is proposed to minimize the transmission energy consumption of the network.
- A parallel data transmission method based on scheduling for the cluster head nodes and sensor nodes is proposed to achieve conflict-free parallel data transmission.
- Replication computing is introduced to the LEACH protocol to reduce the signaling in the clustering and transmission phases.

The rest of this paper is organized as follows: in Section 2, the works related to cluster-based underwater routing protocols are reviewed. In Section 3, the improved LEACH protocol for cluster-based UASNs is proposed. Section 4 presents and analyzes the simulation results. Finally, Section 5 concludes the paper.

## 2. Related Work

In the underwater environment, the batteries of underwater sensor nodes cannot be replaced regularly. Therefore, one of the most important factors in underwater routing protocols is energy limitation. Clustering is a common and effective technique for saving energy. Many cluster-based underwater routing protocols have been proposed for energy conservation. These protocols can be divided into two categories based on the application scenarios, i.e., two-dimensional (2D) and three-dimensional (3D) UASN protocols. Two-dimensional UASNs primarily operate in horizontal planes and are typically used in shallower waters, such as offshore areas, lakes, or rivers. Two-dimensional UASN protocols focus on efficient routing and communication within the horizontal plane. The multi-layer cluster-based energy-efficient (MLCEE) protocol addressed load imbalance and energy consumption in 2D UASN by layering the network and using Bayesian probability for cluster head selection [9]. The quality-of-service (QoS)-aware evolutionary cluster-based routing protocol (QERP) optimized the relay node selection based on traffic and energy consumption [10]. Another 2D UASN protocol, Q-learning and data-priority-based routing protocol with dynamic computing cluster head (QD-DCR), used Q-learning and data prioritization to dynamically select cluster heads and balance energy distribution [11]. However, these protocols often do not fully consider the unique challenges of underwater environments, such as acoustic attenuation and multipath propagation.

Three-dimensional UASNs suitable for deep-water applications cover all three dimensions and must contend with issues such as node localization, dynamic topology, and varying depth. The joint clustering and routing protocol (JCRP) for 3D UASN selects cluster heads based on residual energy and the weighted cost of network connectivity [12]. The cellular-clustering-based interference-aware data transmission protocol (CIDP) for 3D undersea exploration uses TDMA scheduling and hierarchical routing to reduce interference and improve data transmission [13]. In [14], a multi-hop energy-efficient routing protocol was proposed to improve the energy efficiency of data distribution, especially for nodes far from surface sinks. However, these protocols do not adequately address the challenges of 3D underwater communication, particularly in terms of energy-efficient data transmission.

The LEACH protocol, which is one of the most classical cluster-based 2D protocols, randomly selects sensor nodes as cluster heads to aggregate and transmit data to a base station in order to optimize energy consumption and extend the network lifetime. The LEACH protocol was first applied to underwater sensor networks in [15]. Since then, many modified LEACH protocols have been proposed for 2D UASNs. In [16], a modified LEACH protocol was proposed to optimize network energy consumption by finding the ideal number of clusters. But its underwater energy propagation model was too simple and ignored factors like acoustic attenuation and multipath propagation effects. In [17], an adaptation of LEACH was proposed, which employed an absorption-losses-related underwater energy propagation model but overlooked some critical factors in underwater communication, such as transmission loss and excess loss. In [18], a novel solution named underwater modified LEACH (UMOD_LEACH) was proposed to improve the transmission phase in clustering. However, it omits propagation delay and an underwater energy model, which are crucial for underwater communication. In [19], an underwater energy model was applied to the LEACH protocol to calculate the network power consumption and update the node energy. But the transmission loss was not considered in this protocol. These existing modifications to the LEACH protocol, while targeting energy optimization in 2D UASNs, often oversimplify the underwater energy propagation model and overlook crucial aspects of underwater communications such as transmission loss and excess loss. And they have limited transmission efficiency due to low parallelism in data transmission. These limitations underscore the need for an improved LEACH protocol that not only incorporates a more accurate underwater energy model but also focuses on optimizing data packet scheduling to ensure efficient and conflict-free parallel data transmission, thereby addressing both energy management and transmission efficiency in underwater communications.

## 3. Improved LEACH Protocol

### 3.1. Network Model

**Figure 1:**
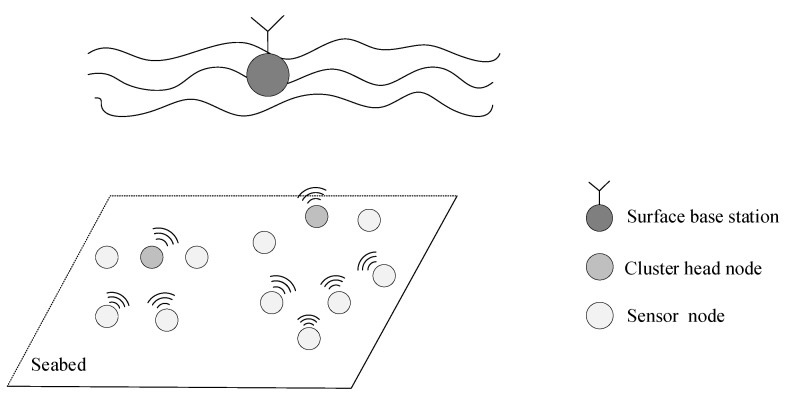
The two-dimensional static network.

In this paper, a 2D static underwater acoustic sensor network is investigated, whose structure is shown in Figure 1. The network consists of a base station, several cluster head nodes, and many sensor nodes. All nodes are anchored to the seabed and autonomously form a cluster-based network. Cluster head nodes are selected using a clustering algorithm. The data collected by each sensor node are converged to the cluster head node and then relayed to the surface base station through vertical links. It is assumed that the network satisfies the following conditions:

(1) All nodes in the network are stationary and uniformly distributed.
(2) All nodes are designed with a homogeneous architecture, allowing each node to function interchangeably as either a sensor node or a cluster head node.
(3) All nodes know the positions and initial energy of other nodes before communication.
(4) The network is partly connected, but all nodes in a cluster can communicate with each other in a half-duplex mode.
(5) All node clocks in the network are synchronized.
(6) During idle periods when there is no communication task, nodes in the network periodically synchronize their residual energy and other information.

### 3.2. Clustering Based on Underwater Energy Propagation Model

#### 3.2.1. Optimization of the Cluster Number

In UASNs, the energy consumption for network operations can be categorized into transmission energy consumption, received energy consumption, and standby energy consumption. Received energy consumption is defined as the energy required by a node to receive and fuse data packets. It is determined by the packet size. Standby energy consumption is defined as the energy consumption of a node in the standby state, which is related to the standby power. These parameters are not controlled by the LEACH protocol, so optimizing received energy and standby energy is beyond the scope of this paper.

Transmission energy consumption is defined as the transmission energy required for a packet to be correctly received by the destination node. It is related by the packet size and the acoustic propagation loss, where the latter can be related to the routing algorithm. The acoustic propagation loss in the water can be calculated by the Marsh–Schulkin model [20] as follows:

(1) $$TL=\left\{\begin{array}{c} 20\mathrm{lg}R+\alpha R+60-K_{L},\;\;\;R<H\\ 15\mathrm{lg}R+\alpha R+\alpha_{r}\left(\frac{R}{H}-1\right)+5\mathrm{lg}H+60-K_{L},\;\;\;H<R<8H\\ 10\mathrm{lg}R+\alpha R+\alpha_{r}\left(\frac{R}{H}-1\right)+10\mathrm{lg}H+60-K_{L},\;\;\;R>8H \end{array}\right.$$

where R denotes the distance from transmitter to receiver, α denotes the seawater absorption coefficient, H denotes the seawater depth, $\alpha_{r}$ denotes the shallow sea attenuation coefficient, and $K_{L}$ denotes the near-field propagation anomaly. In (1), α, *H*, $\alpha_{r}$, and $K_{L}$ are not controllable in the LEACH protocol, so R is the key parameter for optimizing the transmission energy consumption. Note that dTL/dR is always non-negative, so *TL* increases with R in all the three cases of the Marsh–Schulkin model, i.e., minimizing the transmission energy consumption is equivalent to minimizing R. In other words, to minimize the energy consumption of network operations, the total transmission distance of all packets throughout the network should be as small as possible. Based on this observation, the optimal number of clusters $K_{opt}$ that minimizes the total transmission distance can be given by:

(2) $$K_{opt}=\underset{K}{argmin}R_{S}$$

where $R_{S}$ denotes the total transmission distance of all packets, and K denotes the number of clusters.

Because the packets sent by the sensor nodes are received and merged by the cluster head nodes before being forwarded to the base station, the total transmission distance is the sum of the distances between all sensor nodes to their cluster head nodes and all cluster head nodes to the base station as follows:

(3) $$R_{S}=\overset{K}{\underset{i=1}{\sum }}\underset{m}{\sum }r_{i}^{m}+\overset{K}{\underset{i=1}{\sum }}r_{i}^{BS}$$

where $r_{i}^{m}$ denotes the Euclidean distance between the *m*th sensor node and the cluster head node within the *i*th cluster, $r_{i}^{BS}$ denotes the distance between the *i*th cluster head node and the based station.

From Equations (2) and (3), it can be seen that $K_{opt}$ is related to the node’s locations. Then, (3) can be expressed as follows [21]:

(4) $$R_{S}=\overset{K}{\underset{i=1}{\sum }}\underset{m}{\sum }r_{i}^{m}+\overset{K}{\underset{i=1}{\sum }}r_{i}^{BS}=\left(N-K\right)\bar{r}+K\bar{r^{BS}}$$

where N denotes the number of underwater sensor nodes and $\bar{r}$ denotes the mean of the distances between the sensor nodes and cluster head nodes in the network as follows:

(5) $$\bar{r}=\frac{1}{N-K}\overset{K}{\underset{i=1}{\sum }}\underset{m}{\sum }r_{i}^{m}$$

where $\bar{r^{BS}}$ denotes the mean of the distances between cluster head nodes and base stations in the network as follows:

(6) $$\bar{r^{BS}}=\frac{1}{K}\overset{K}{\underset{i=1}{\sum }}r_{i}^{BS}$$

Assuming that N underwater sensor nodes are uniformly distributed in a square with the length of l, when N is relatively large, (5) can be approximated using integral calculus to represent the expected value of $\bar{r}$, since the discrete summation converges to a continuous distribution by the law of large numbers. So, (5) can be approximated as follows:

(7) $$\bar{r}\approx E\left(r_{i}^{m}\right)=\int_{0}^{2\pi }\int_{0}^{\sqrt{\frac{l^{2}}{K\pi }}\;}r\rho_{1}\left(r,\theta \right)\cdot rdrd\theta =\frac{2\pi }{3}\cdot \frac{l^{3}}{{\left(K\pi \right)}^{\frac{3}{2}}}\cdot \frac{N}{l^{2}}=\frac{2Nl}{3\sqrt{\pi }}\cdot \frac{1}{K^{\frac{3}{2}}}$$

where $\rho_{1}\left(r,\theta \right)$ denotes the probability density function of the sensor node positions, and $\rho_{1}\left(r,\theta \right)=\frac{N}{l^{2}}$ when this distribution is uniform.

Similarly, (6) can be approximated as follows:

(8) $$\bar{r^{BS}}\approx E\left(r_{i}^{BS}\right)=\int_{0}^{2\pi }\int_{0}^{\sqrt{\frac{l^{2}}{\pi }}\;}r\rho_{2}\left(r,\theta \right)\cdot rdrd\theta =\frac{2\pi }{3}\cdot \frac{l^{3}}{\pi^{\frac{3}{2}}}\cdot \frac{K}{l^{2}}=\frac{2l}{3\sqrt{\pi }}\cdot K$$

where $\rho_{2}\left(r,\theta \right)$ denotes the probability density function of the cluster head node positions, and $\rho_{2}\left(r,\theta \right)=\frac{K}{l^{2}}$ when this distribution is uniform.

Substituting (7) and (8) into (4), $R_{S}$ is given by:

(9) $$R_{S}\approx N\cdot \left(\frac{2Nl}{3\sqrt{\pi }}\cdot \frac{1}{K^{\frac{3}{2}}}\right)+K\cdot \left(\frac{2l}{3\sqrt{\pi }}\cdot K\right)=\frac{2N^{2}l}{3\sqrt{\pi }}\cdot \frac{1}{K^{\frac{3}{2}}}+\frac{2l}{3\sqrt{\pi }}\cdot K^{2}$$

To obtained $K_{opt}$, let $\frac{dR_{s}}{dK}=0$, i.e.,:

(10) $${\left.\frac{dR_{s}}{dK}\right|}_{K=K_{opt}}=-\frac{N^{2}l}{\sqrt{\pi }}\cdot \frac{1}{K^{\frac{5}{2}}}+\frac{4l}{3\sqrt{\pi }}\cdot K=0$$

 $K_{opt}$ is given by:

(11) $$K_{opt}=\sqrt[{7}]{\frac{9N^{4}}{16}}$$

#### 3.2.2. Improved Clustering Algorithm

According to the above discussion, the energy consumption can be reduced by minimizing the total communication distance of nodes in the network, which can be implemented by optimizing the cluster number. Therefore, we propose an improved clustering algorithm by adding a cluster number optimization step into the conventional K-means clustering algorithm. Assuming that the position and the initial energy of each node are known, the proposed clustering algorithm is described in Algorithm 1.

**Algorithm 1**: Improved clustering algorithm1: Initialize the number and location distribution of nodes;2: Initialize the residual energy of each node and the set C of cluster head nodes as empty;3: Compute the network optimum cluster number $K_{opt}$;4: Each node obtains the minimum value of residual energy $E_{m}$ of cluster head nodes;5: **for** each iteration number e **do**6:  Clear the set C;7:  **for** each node i **do**8:   **if** $E_{i}>0.5E_{m}$ **then**9:    Include node i in C for this round.10:   **end if**11:  **end for**12:  Cluster nodes into $K_{opt}$ clusters with the K-means algorithm; nodes in C are used as cluster head nodes;13:  Each node obtains the minimum value of residual energy $E_{m}$ of cluster head nodes;14: **end for**

### 3.3. Parallel Transmission Based on Scheduling for Cluster Head Nodes and Sensor Nodes

Since the transmission rate between two nodes in underwater acoustic networks is usually very low due to the narrow bandwidth of the underwater acoustic channel, increasing the transmission parallelism among network nodes is one of the most effective ways to improve the performance of underwater acoustic networks [22]. Parallel transmission in the underwater environment can be implemented by using time division multiple access (TDMA), code division multiple access (CDMA), and frequency division multiple access (FDMA), among which TDMA is the most widely used method due to its convenience and ability to exploit the long propagation delay. In this context, our study specifically adopts the TDMA approach for transmission scheduling. This choice was motivated by its inherent ability to exploit temporal parallelism through differential propagation delay among nodes, a feature that is critically dependent on strategic transmission scheduling. In cluster-based networks, data transmission involves two phases: sensor nodes and cluster head nodes. Effective scheduling requires careful timing coordination between these entities to ensure efficient data exchange.

#### 3.3.1. Scheduling for Cluster Head Nodes

**Figure 2:**
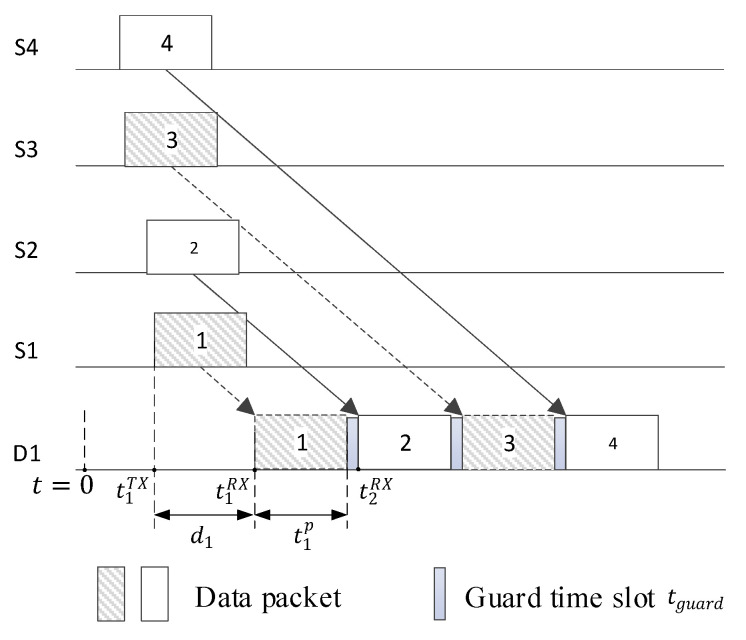
Scheduling for cluster head nodes.

The ideal scheduling for cluster head nodes is that the packets sent by the cluster head nodes are received sequentially by the base station with minimum intervals between packets, as shown in Figure 2, and the optimal times of arrival of the Q cluster head nodes should be given by:

(12) $$t_{i}^{RX}=\left\{\begin{array}{c} d_{1},\;\;\;\;\;\;\;\;\;\;\;\;\;\;\;i=1\\ t_{i-1}^{RX}+t_{bs}^{p}+t_{guard},\;\;i=2,\ldots ,Q \end{array}\right.$$

where $d_{1}$ denotes the transmission delay from the first cluster head node to the base station, $t_{i}^{RX}$ denotes the arrival time of the packet from the *i*th cluster head node to the base station, $t_{bs}^{p}$ denotes the duration of the base station receiving a packet, and $t_{guard}$ denotes the guard time slot, respectively. And $t_{guard}$ is designed to account for the uncertainty in the propagation delay estimates, ensuring that even if there is a slight delay in the signal reaching its destination, it will not interfere with the subsequent transmission slot. Then, the transmission time $t_{i}^{TX}$ of the *i*th cluster head node is given by:

(13) $$t_{i}^{TX}=t_{i}^{RX}-d_{i},\;\;i=1,\ldots ,Q$$

where $d_{i}$ denotes the transmission delay of the cluster head node Si. Note that if $d_{i}$ is large, $t_{i}^{TX}$ may be negative in (13). In this case, the actual transmission time of each node should be adjusted as follows:

(14) $$t_{i}^{TX\prime }=t_{i}^{TX}-min\left(t_{j}^{TX}\right)\;\;i,j=1,\ldots ,Q$$

#### 3.3.2. Scheduling for Sensor Nodes

When a node transmits a data packet, the acoustic wave propagates outward in a spherical pattern. It is possible that the transmission of a node will affect the reception of other nodes in adjacent clusters. It is assumed that when a destination node is within a circle with a radius of $a_{d}r$ centered on the source node, its reception will be interfered with by this source node, and vice visa, where r is the distance from the source node to the destination node and $a_{d}$ is a threshold. There are three types of conflicts that should be considered when scheduling the transmission time of the *j*th node in the *i*th cluster.

The first type is the intra-cluster conflicts, which are caused by the scheduled nodes in the same cluster. A data conflict occurs when the completion time for the *i*th cluster head to receive data from the *j*th sensor node exceeds the arrival time to receive data from the *l*th sensor node, and vice versa. Therefore, for the *j*th node in the *i*th cluster, the transmission time that will cause conflict with the *l*th node in the *i*th cluster at the *i*th cluster head node satisfies the following inequalities:

(15) $$t_{j}^{i}+d_{ij}^{i}+t_{guard}+t^{p}\geq t_{l}^{i}+d_{il}^{i}\;\mathrm{and}\;t_{j}^{i}+d_{ij}^{i}\leq t_{l}^{i}+d_{il}^{i}+t_{guard}+t^{p}$$

where 1≤l≤j−1. $t_{j}^{i}$ and $t_{l}^{i}$ denote the transmission times of the *j*th and *l*th sensor nodes in the *i*th cluster. $d_{ij}^{i}$ and $d_{il}^{i}$ denote the propagation delays from the *j*th and *l*th sensor nodes to the *i*th cluster head node. $t^{p}$ denotes the duration of the cluster head node receiving a packet. So, $t_{j}^{i}$ that will cause conflict with the transmission of the first to the (*j* − 1)th node in the *i*th cluster belongs to the set of

(16) $$U_{i,j}^{ita}=\overset{j-1}{\underset{l=1}{\cup }}\left[t_{l}^{i}+d_{il}^{i}-d_{ij}^{i}-t_{guard}-t^{p},t_{l}^{i}+d_{il}^{i}-d_{j}^{i}+t_{guard}+t^{p}\right]$$

The second type is the inter-cluster conflicts caused by the current transmission at the adjacent cluster head nodes. It is assumed that there are *M* cluster head nodes that are interfered with by the transmission of the *j*th node in the *i*th cluster. A data conflict occurs at the *m*th adjacent cluster head node if the completion time for the *m*th cluster head node to receive data from the *l*th sensor node within the same *m*th cluster exceeds the arrival time of the data sent by the *j*th sensor node from the *i*th cluster. Similarly, a data conflict is also triggered at the *m*th cluster head node if the completion time for the *m*th cluster head node to receive data from the *j*th sensor node in the *i*th cluster exceeds the arrival time of the data from the *l*th sensor node in the *m*th cluster to its cluster head node. Then, $t_{j}^{i}$ that will cause conflict with the transmission of the *l*th sensor node in the *m*th cluster at the *m*th cluster head node satisfies the following inequalities:

(17) $$t_{j}^{i}+d_{mj}^{i}+t_{guard}+t^{p}\geq t_{l}^{m}+d_{ml}^{m}\;\mathrm{and}\;t_{j}^{i}+d_{mj}^{i}\leq t_{l}^{m}+d_{ml}^{m}+t_{guard}+t^{p}$$

So, $t_{j}^{i}$ that will cause conflicts at the *m*th adjacent cluster head node belongs to the set of:

(18) $$U_{i,j}^{iten}=\overset{M}{\underset{m=1}{\cup }}\overset{J_{m}}{\underset{l=1}{\cup }}\left[t_{l}^{m}+d_{ml}^{m}-d_{mj}^{i}-t_{guard}-t^{p},t_{l}^{m}+d_{ml}^{m}-d_{mj}^{i}+t_{guard}+t^{p}\;\right]$$

where $J_{m}$ is the number of sensor nodes in the *m*th cluster.

The third type is the inter-cluster conflicts caused by the transmission of the sensor nodes in adjacent clusters at the *i*th cluster head node. It is assumed that there are *N* adjacent cluster transmissions that will interfere with the reception of the *i*th cluster head nodes. A data conflict is induced at the *i*th cluster head node if the completion time of data reception from the *j*th sensor node within the same *i*th cluster exceeds the arrival time of the data transmitted by the *l*th sensor node from the *n*th cluster. Correspondingly, a data conflict is also induced if the completion of data reception from the *l*th sensor node in the *n*th cluster is delayed beyond the arrival of data from the *j*th sensor node in the *i*th cluster at the respective cluster head node. Then, $t_{j}^{i}$ that will cause conflict with the transmission of the *l*th sensor node in the *n*th adjacent cluster at the *i*th cluster head node satisfies the following inequalities:

(19) $$t_{j}^{i}+d_{ij}^{i}+t_{guard}+t^{p}\geq t_{l}^{n}+d_{il}^{n}\;\mathrm{and}\;t_{j}^{i}+d_{ij}^{i}\leq t_{l}^{n}+d_{il}^{n}+t_{guard}+t^{p}$$

So, $t_{j}^{i}$ that will cause conflicts with sensor nodes in adjacent clusters at the *i*th cluster head node belongs to the set of:

(20) $$U_{i,j}^{iteh}=\overset{N}{\underset{n=1}{\cup }}\overset{J_{n}}{\underset{l=1}{\cup }}\left[t_{l}^{n}+d_{il}^{n}-d_{ij}^{i}-t_{guard}-t^{p},t_{l}^{n}+d_{il}^{n}-d_{ij}^{i}+t_{guard}+t^{p}\;\right]$$

where $J_{n}$ is the number of sensor nodes that will cause conflicts at the *i*th cluster head nodes in the *n*th cluster.

Let ${\bar{U}}_{i,j}=\bar{\left(U_{i,j}^{ita}\cup U_{i,j}^{iten}\cup U_{i,j}^{iteh}\right)}$; then, the conflict-free transmission time of the *j*th node in the *i*th cluster can be given by

(21) $$t_{j}^{i}=\underset{t_{j}^{i}\in {\bar{U}}_{i,j}}{min}\left(max\left(t_{j}^{i},t_{k}\right)-min\left(t_{j}^{i},t_{k}\right)\right)$$

where $t_{k}$ denotes the transmission time of scheduled nodes in the network.

Finally, all transmission times are aligned to avoid negatives when the scheduling of all sensor nodes is complemented as:

(22) $$t_{k}^{\prime }=t_{k}-min\left(t_{k}\right)$$

The proposed scheduling for sensor nodes consists of the following steps:

1. Schedule the transmission times of all sensor nodes in the first cluster.
2. Find out the nodes in the *i*th cluster that will interfere with the reception of the first to the (*i* − 1)th cluster head nodes and the nodes in the first to the (*i* − 1)th clusters that will interfere with the reception of the *i*th cluster head node according to their locations.
3. Obtain the transmission schedules of the first to the (*i* − 1)th clusters.
4. For the *j*th node in the *i*th cluster, calculate its transmission time that does not cause conflicts at the *i*th and the scheduled adjacent cluster head nodes while minimizing the total transmission time.
5. Repeat step 4 until all the transmission times of the nodes in the *i*th cluster are scheduled. Then, schedule the transmission time of the next cluster.
6. Repeat steps 2~5 until all the nodes in the network are scheduled.

### 3.4. LEACH Protocol Based on Replication Computing

#### 3.4.1. Principle of Replication Computing

**Figure 3:**
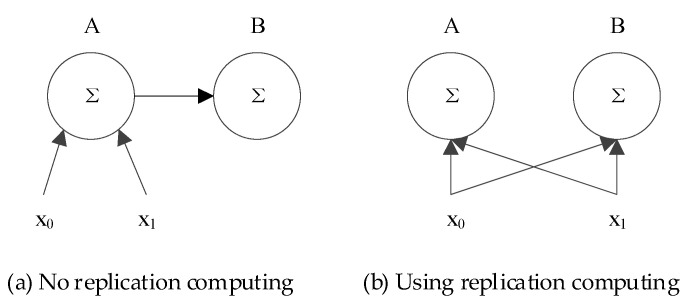
An example of using replication computing to reduce communication in parallel computing.

Replication computing is a technique that uses unnecessary and redundant computations in parallel computing to reduce communication or execution time [23]. Figure 3 illustrates an example of leveraging replication computing to reduce communication in parallel computing. In this example, the task involves summing two input variables $x_{0},x_{1}$ and storing the result in processors A and B. In Figure 3a, processor A first acquires the inputs $x_{0}$ and $x_{1}$ via communication, computes their sum, and then transmits the result to B. Conversely, Figure 3b depicts a scenario where both inputs $x_{0}$ and $x_{1}$ are broadcasted to A and B. Then, each processor independently computes the sum. This example shows that, in contexts where broadcasting incurs no extra communication cost (e.g., bus structures or one-hop networks), the latter approach reduces one instance of inter-processor communication at the cost of duplicating computational tasks. This advantage makes replication computing attractive to underwater acoustic communication that is characterized by a large propagation delay.

#### 3.4.2. Protocol Implementation

In the proposed LEACH protocol, the clustering and scheduling algorithms all need to know a lot of information about the states of other nodes, like the residual energy and the scheduled transmission times. Exchanging this information needs extensive information exchange, which will decrease the performance of the network significantly. So, replication computing is introduced to the proposed LEACH protocol to reduce the signaling.

In general, an operation suitable for replication computing should satisfy the following conditions:

(1) The inputs of the operation are variable.
(2) Multiple nodes must know the operation results.
(3) The operation is deterministic.

The clustering and scheduling operations satisfy all the conditions required by the replication computing. For the clustering, the residual energy of each node, which is the input of the clustering algorithm, is changed every round. All nodes must know the clustering results. The clustering method proposed in Section 3.2 is deterministic. For the scheduling, the destination nodes, i.e., the cluster head nodes, which are the input of the scheduling algorithm, change every round. Each sensor node must know the transmission schedules of other nodes to avoid collision. The scheduling method proposed in Section 3.3 is deterministic. Hence, replication computing can be used to reduce the signaling in the clustering and transmission phases of the proposed LEACH protocol.

The basic idea of applying replication computing to the proposed LEACH protocol is that each node can calculate the states of all other nodes in the whole network independently through replication computing if the initial states of other nodes are known, including the residual energies and transmission schedules. Based on this idea, the steps of the proposed protocol are as follows:

1. Initialize a table in each node that contains information about the location, initial energy, and schedule order of all nodes in the network.
2. Activate the network by transitioning from standby to active mode for the communication task. Each node updates the residual energies of all nodes in the network according to the following formula:
  (23) $$E_{i}^{\prime }=E_{i}-E_{standby}$$
  where $E_{i}^{\prime }$ is the updated residual energy of the *i*th node, $E_{i}$ is the initial residual energy, and $E_{standby}$ is the energy consumption during the standby mode.
3. Each node independently computes a consistent network clustering result using the proposed clustering algorithm through replication computing.
4. Each node establishes an identical packet scheduling table using the proposed scheduling algorithm through replication computing.
5. Sensor nodes and cluster head nodes send packets at predetermined times.
6. Each node updates the residual energies of all nodes in the network through replication computing. The energy update process differs between sensor nodes and cluster head nodes as follows:
  For sensor nodes:
  (24) $$E_{i}^{n\prime }=E_{i}^{n}-E_{i}^{nt}$$
  where $E_{i}^{n\prime }$ is the updated residual energy of the *i*th sensor node, $E_{i}^{n}$ is its initial residual energy, and $E_{i}^{nt}$ is the energy consumed by the *i*th sensor node during transmission.
  For cluster head nodes:
  (25) $$E_{i}^{h\prime }=E_{i}^{h}-E_{i}^{ht}-E_{i}^{hc}$$
  where $E_{i}^{h\prime }$ is the updated residual energy of the *i*th cluster head node, $E_{i}^{h}$ is its initial residual energy, $E_{i}^{ht}$ is the energy consumed by the *i*th cluster head node during transmission, and $E_{i}^{hc}$ is the energy consumed by the *i*th cluster head node during data reception.
7. Continue with the next round of communication or enter standby mode.

Note that nodes in the network will periodically synchronize their residual energy and other information during idle periods when there is no communication task to avoid accumulated calculation errors of replication calculation.

## 4. Simulations and Results

### 4.1. Simulation Setup

To evaluate the performance of the proposed protocol, simulations were conducted with the original LEACH protocol [5], the KMMDA algorithm [24], the improved clustering algorithm proposed in Section 3.2, which has the same transmission scheduling algorithm as the original LEACH protocol, and the proposed protocol without using replication computing, respectively. The experiments were based on the NS3 simulation platform. The experimental parameters are given in Table 1.

### 4.2. Simulation Metrics

The performance of the proposed protocol was evaluated in terms of the average residual energy, the average number of surviving nodes, the average round when the first death node occurs, and the number of packets received by the base station.

The average residual energy is a measure of network energy consumption. When the residual energy of a node falls below a threshold, the node is unable to join a cluster and sends/receives packets normally so that becomes a dead node. The average residual energy is calculated by:

(26) $$\mathrm{Average}\;\mathrm{residual}\;\mathrm{energy}=\frac{\sum E_{i}^{n}}{N\cdot N_{SR}}$$

where $E_{i}^{n}$ denotes the residual energy of node i at the *n*th round. $N_{SR}$ denotes the number of simulation groups.

The average number of surviving nodes is also a measure of network energy consumption and is calculated by:

(27) $$\mathrm{Average}\;\mathrm{number}\;\mathrm{of}\;\mathrm{surviving}\;\mathrm{nodes}=\frac{\sum N_{s}^{n}}{N_{SR}}$$

where $N_{s}^{n}$ denotes the number of nodes in the network with residual energy greater than 0 at the *n*th round.

The average round when the first death node occurs is calculated by:

(28) $$\mathrm{Average}\;\mathrm{round}\;\mathrm{when}\;\mathrm{the}\;\mathrm{first}\;\mathrm{death}\;\mathrm{node}\;\mathrm{occurs}=\frac{\sum R_{d}}{N_{SR}}$$

where $R_{d}$ denotes the round when the first death node occurs.

The number of packets received by the base station can be obtained directly by experimental simulation.

### 4.3. Simulation Results

#### 4.3.1. Proposed Protocol versus LEACH and KMMDA

**Figure 4:**
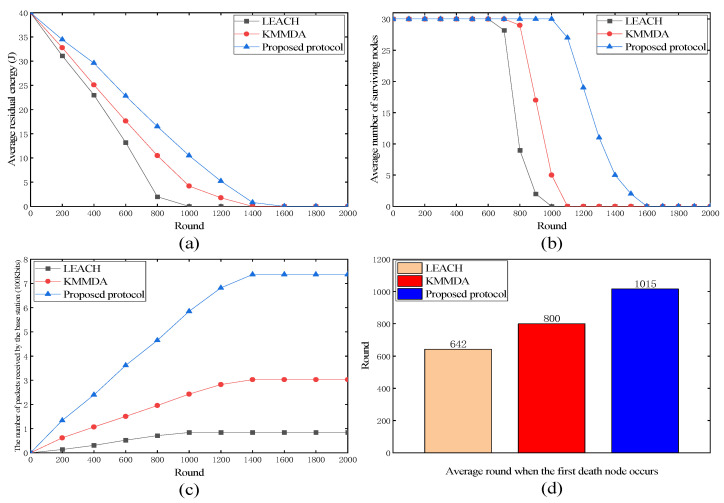
Performance of LEACH, KMMDA, and proposed protocol. (**a**) Average residual energy; (**b**) average number of surviving nodes; (**c**) the number of packets received by the base station; (**d**) average round when the first death node occurs.

As shown in Figure 4a, the proposed protocol showed a slower decline in the average residual energy over time, indicating more efficient energy usage compared to LEACH and KMMDA. As shown in Figure 4b, the proposed protocol maintained a higher number of surviving nodes for a longer period, suggesting that it better preserved the node lifetime and prolonged the network functionality. As shown in Figure 4c, the proposed protocol resulted in a consistently higher number of packets received by the base station, indicating a superior data transmission capability and network throughput. As shown in Figure 4d, the bar chart illustrates that the first node death occurred significantly later in the proposed protocol, demonstrating its effectiveness in energy distribution and management.

In summary, the proposed protocol outperformed LEACH and KMMDA in these four metrics, leading to enhanced network performance and reliability.

#### 4.3.2. Comparative Analysis of Protocol Improvements

**Figure 5:**
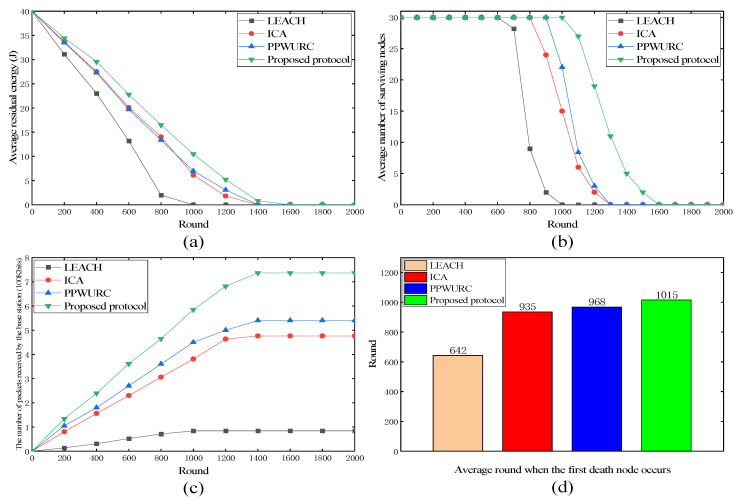
Performance of LEACH, ICA, PPWURC, and proposed protocol. (**a**) Average residual energy; (**b**) average number of surviving nodes; (**c**) the number of packets received by the base station; (**d**) average round when the first death node occurs.

A comparison of the improved parts of the proposed protocol is shown in Figure 5, where “ICA” denotes the improved clustering algorithm and “PPWURC” denotes the proposed protocol without using replication computing.

The proposed protocol showed significant improvements over LEACH, ICA, and PPWURC, as depicted in Figure 5, offering enhanced network performance. As shown in Figure 5a, the proposed protocol maintained higher residual energy levels over an extended number of rounds compared to the others, indicating more efficient energy usage. As shown in Figure 5b, the proposed protocol sustained a greater number of surviving nodes for longer durations, suggesting superior node longevity and network stability. As shown in Figure 5c, the proposed protocol achieved a higher number of packets received by the base station, indicating an improved data transmission capability. As shown in Figure 5d, the first node death occurred substantially later in the proposed protocol, demonstrating its effective energy management and improved node lifetime.

The advances of the proposed protocol are attributed to the integration of an improved clustering algorithm from ICA, parallel transmission scheduling from PPWURC, and the implementation of replication computing, which collectively contribute to its superior performance.

#### 4.3.3. Performance Evaluation under Different Node Densities

**Figure 6:**
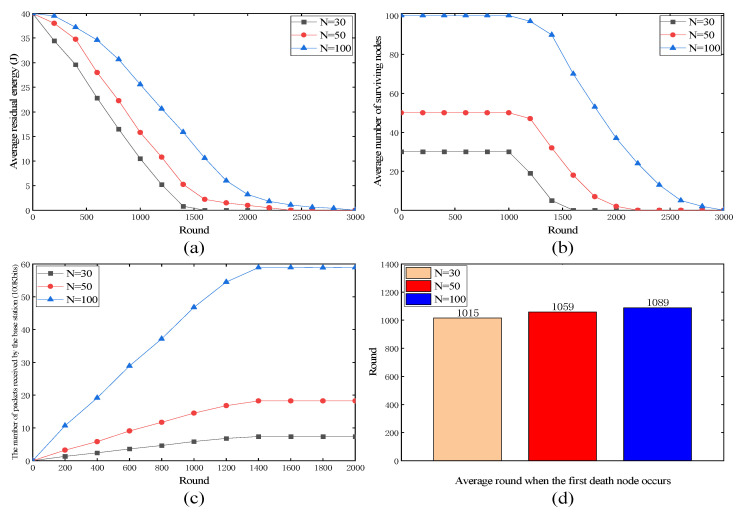
Performance of proposed protocol under different node densities. (**a**) Average residual energy; (**b**) average number of surviving nodes; (**c**) the number of packets received by the base station; (**d**) average round when the first death node occurs.

Figure 6 shows the performance of the proposed protocol at different node densities. Figure 6a shows that at higher node densities, the average residual energy of the network depleted more slowly, suggesting that the proposed protocol effectively managed the collective energy resource among a larger number of nodes. As shown in Figure 6b, the rate of the decrease in the number of surviving nodes was relatively slower in denser networks. However, the proposed protocol maintained a reasonable number of nodes across all node densities. This indicates that it adapted well to changes in the node density. As shown in Figure 6c, there was a noticeable increase in the number of packets received as the node density increased. This indicates that the proposed protocol scales efficiently with larger network sizes, resulting in improved data throughput. As shown in Figure 6d, the proposed protocol demonstrated the ability to delay the occurrence of the first node death as the node density increased. This implies that it effectively handles higher node densities, allowing for longer network operation.

In summary, the proposed protocol exhibits robust performance over varying node densities. It effectively manages energy consumption, adapts to changes in node density, delays node failures, and scales well with increasing network sizes, ultimately improving data transmission efficiency.

## 5. Conclusions

In this paper, we proposed an improved LEACH protocol for cluster-based UASNs where the cluster number is optimized with an underwater energy propagation model to reduce the network’s energy consumption, and a transmission scheduling algorithm was also proposed to achieve conflict-free parallel data transmission. Furthermore, the integration of replication computing into the proposed protocol allows the network nodes to autonomously execute a deterministic algorithm that determines their clusters and conflict-free transmission times for each communication round. This approach significantly reduces the energy consumption of the network during signaling interactions, further increasing its lifetime. The proposed protocol was simulated and compared with the existing methods in NS3, and the experimental results proved the viability of the proposed protocol.

However, this paper focused exclusively on stationary, two-dimensional underwater acoustic sensor networks, leaving the dynamics of mobile nodes and three-dimensional node distribution unexplored. In our focused study, we focused on the path length in acoustic energy transfer, recognizing its significant impact on the efficiency within these specific network parameters. The path length was found to be a critical factor in the effective energy transfer for the considered scenarios. Nevertheless, a comprehensive understanding requires the consideration of additional factors such as medium properties, signal frequency, and environmental disturbances, which, while recognized, were not the primary focus of our current research. Future studies should extend both the proposed clustering algorithm and the parallel transmission scheduling algorithm to scenarios involving mobile nodes and nodes arranged in a three-dimensional space, incorporating these broader aspects for a more thorough exploration of acoustic energy transfer. Additionally, while this paper examined UASNs within a two-layer centralized framework, future research could delve into more complex network structures, such as distributed or multi-hop configurations, to provide a comprehensive analysis and understanding of these specific network scenarios.

## Figures and Tables

**Table 1:** Parameters of experimental simulation.

Parameter	Value
Number of nodes *N*	30
Maximum number of iterations $e_{max}$	2000
Network communication rounds $e_{p}$	2000
Node initial energy $E_{0}$	40 J
Length of water body l	150 m
Node depth H	50 m
Packet reception minimum sound level $I_{0}$	1 μw
Non-cluster header packet size $b^{CH}$	50 bits
Cluster header packet maximum $b^{BS}$	500 bits
Node receives 1 bit of data energy consumption $e^{\prime RX}$	$5\times 10^{-5}\;J$
Guard time slot $t_{guard}$	0.2 s
Underwater sound velocity v	1500 m/s

## Data Availability

Data are contained within the article.

## References

[1] Akyildiz I.F., Pompili D., Melodia T. (2005). Underwater acoustic sensor networks: Research challenges. Ad Hoc Netw..

[2] Luo H., Wu K., Ruby R., Hong F., Ni L.M. (2017). Simulation and Experimentation Platforms for Underwater Acoustic Sensor Networks: Advancements and Challenges. ACM Comput. Surv..

[3] Luo J., Fan L., Wu S., Yan X. (2017). Research on localization algorithms based on acoustic communication for underwater sensor networks. Sensors.

[4] Pompili D., Akyildiz I.F. (2009). Overview of networking protocols for underwater wireless communications. IEEE Commun. Mag..

[5] Heinzelman W.R., Chandrakasan A., Balakrishnan H. Energy-efficient communication protocol for wireless microsensor networks. Proceedings of the 33rd Annual Hawaii International Conference on System Sciences.

[6] Younis O., Fahmy S. (2004). HEED: A hybrid, energy-efficient, distributed clustering approach for ad hoc sensor networks. IEEE Trans. Mob. Comput..

[7] Yi S., Heo J., Cho Y., Hong J. (2007). PEACH: Power-efficient and adaptive clustering hierarchy protocol for wireless sensor networks. Comput. Commun..

[8] Qing L., Zhu Q., Wang M. (2006). Design of a distributed energy-efficient clustering algorithm for heterogeneous wireless sensor networks. Comput. Commun..

[9] Khan W., Wang H., Anwar M.S., Ayaz M., Ahmad S., Ullah I. (2019). A Multi-Layer Cluster Based Energy Efficient Routing Scheme for UWSNs. IEEE Access.

[10] Faheem M., Tuna G., Gungor V.C. (2018). QERP: Quality-of-Service (QoS) Aware Evolutionary Routing Protocol for Underwater Wireless Sensor Networks. IEEE Syst. J..

[11] Tu S., Zhu X., Chen Y., Xu X. A Q-Learning and Data Priority-Based Routing Protocol with Dynamic Computing Cluster Head for Underwater Acoustic Sensor Networks. Proceedings of the 2022 IEEE International Conference on Signal Processing, Communications and Computing (ICSPCC).

[12] Dhongdi S., Bhandari A., Singh J., Kachhadia S., Joshi V. Joint clustering and routing protocol for 3-D underwater acoustic sensor network. Proceedings of the 2018 Tenth International Conference on Ubiquitous and Future Networks (ICUFN).

[13] Zhang J., Cai M., Han G., Qian Y., Shu L. (2020). Cellular clustering-based interference-aware data transmission protocol for underwater acoustic sensor networks. IEEE Trans. Veh. Technol..

[14] Liu X., Zhou F. Energy-Efficient Routing Protocol Based on Data Dissemination for Underwater Wireless Sensor Network. Proceedings of the OCEANS 2023-Limerick.

[15] Li X., Fang S.-L., Zhang Y.-C. The study on clustering algorithm of the underwater acoustic sensor networks. Proceedings of the 2007 14th International Conference on Mechatronics and Machine Vision in Practice.

[16] Zhang Y., Sun H. A clustered routing protocol for underwater wireless sensor networks. Proceedings of the 2015 34th Chinese Control Conference (CCC).

[17] Mansouri D., Ioualalen M. Adapting LEACH algorithm for underwater wireless sensor networks. Proceedings of the Eleventh International Multi-Conference on Computing in the Global Information Technology.

[18] Alhazmi A.S., Moustafa A.I., AlDosari F.M. Energy aware approach for underwater wireless sensor networks scheduling: UMOD_LEACH. Proceedings of the 2018 21st Saudi Computer Society National Computer Conference (NCC).

[19] Rizvi H.H., Khan S.A., Enam R.N. (2022). Clustering base energy efficient mechanism for an underwater wireless sensor network. Wirel. Pers. Personal. Commun..

[20] Schulkin M., Mercer J.A. (1985). Colossus Revisited: A Review and Extension of the Marsh-Schulkin Shallow Water Transmission Loss Model.

[21] Heinzelman W.B., Chandrakasan A.P., Balakrishnan H. (2002). An application-specific protocol architecture for wireless microsensor networks. IEEE Trans. Wirel. Commun..

[22] Zhang J., Hu Z., Xiong Y., Ning G. (2020). A collision-free hybrid MAC protocol based on pipeline parallel transmission for distributed multi-channel underwater acoustic networks. Electronics.

[23] Zhang J., Lai H., Xiong Y. (2019). Concurrent transmission based on distributed scheduling for underwater acoustic networks. Sensors.

[24] Peng W., Edwards D.J. K-means like minimum mean distance algorithm for wireless sensor networks. Proceedings of the 2010 2nd International Conference on Computer Engineering and Technology.

